# Bonding Behavior of Repair Material Using Fly-Ash/Ground Granulated Blast Furnace Slag-Based Geopolymer

**DOI:** 10.3390/ma12101697

**Published:** 2019-05-24

**Authors:** Wen-Ten Kuo, Ming-Yao Liu, Chuen-Ul Juang

**Affiliations:** Department of Civil Engineering, National Kaohsiung University of Science and Technology, Kaohsiung 807, Taiwan; qwerty125679@gmail.com (M.-Y.L.); vivian820830@gmail.com (C.-U.J.)

**Keywords:** fly ash/ground-granulated blast-furnace slag geopolymer, repair material, bond strength, repair mechanism

## Abstract

Fly ash/ground-granulated blast-furnace slag geopolymer (FGG) contains reaction products with a high volume of Ca, hydrated CaSiO_3_, and hydrated AlCaSiO_3_. These compounds enable the filling of large air voids in a structure, thus increasing compactness. Therefore, FGG is a more effective repair material to stabilize structures and can function as a sealing and insulating layer. This study used FGG as the repair material for concrete with ground-granulated blast-furnace slag (GGBFS) as the main cement material. The bond strength of the repair was discussed from different aspects, including for fly-ash substitution rates of 0%, 10%, 20%, and 30% and for liquid–solid ratios of 0.4 and 0.5. The slant shear test, and the split tensile test were employed in this analysis. Moreover, acoustic emission (AE) and scanning electron microscopy were used to confirm the damage modes and microstructural characteristics of these repairs. The results revealed that when the liquid–solid ratio increased from 0.4 to 0.5, the slant shear strength of the repaired material decreased from 36.9 MPa to 33.8 MPa, and the split tensile strength decreased from 1.97 MPa to 1.87 MPa. The slant shear test and split tensile test demonstrated that the repair material exhibited the highest effectiveness when the fly-ash substitution was 10%, and revealed that the repair angle directly affected the damage modes. The AE technique revealed that the damage behavior pattern of the FGG repair material was similar to that of Portland concrete. The microstructural analysis revealed that the FGG–concrete interphase contained mostly hydration products, and based on energy-dispersive X-ray spectroscopy (EDX), the compactness in the interphase and bond strength increased after the polymerization between the geopolymer and concrete. This indicated that the geopolymer damage mode was highly related to the level of polymerization.

## 1. Introduction

Repair materials play a crucial role in extending the lifespan and durability of concrete structures to ensure environmental protection, cost reduction, and enhanced safety. During use, repair materials should be able to sustain the stress change induced by changes in volume in addition to chemical and electrochemical factors, thereby preventing stress relaxation or deterioration and providing the structure with longer durability [1]. The interface between repair materials and the existing concrete structure is known as the interphase, which constitutes the weakest area between repair materials and this structure. Repair materials are crucial to the effectiveness of a repair, as they affect the performance of the interphase [2]. Such materials are commonly used in the engineering industry to repair larger cracks and defective parts or holes and restore the damaged concrete surface to its original appearance. To ensure that the physical properties and colors of repair materials are similar to those of the original concrete, concrete and concrete-like materials are commonly used as repair materials, such as cement mortar, non-shrinkage cement, non-shrinkage grout, latex mortar, and lightweight resin mortar. In Taiwan, concrete-like repair materials commonly used in construction projects have high compatibility with the original concrete, but are ineffective in alleviating the high level of dry shrinkage and poor anti-permeability of the original concrete. Consequently, cracks, carbonization, corrosion of steel bars, and insufficient bond strength are frequently observed in repaired concrete structures [3]. Moreover, repaired structures often exhibit distinct mechanical properties from those of the original structures, thus leading to ineffective repairs and resource wastage. Li et al. [2,4] compared cement-based repair materials with pure cement mortar, expanded adhesive, and fly-ash mortar, and discovered that when fly ash/ground-granulated blast-furnace slag geopolymer (FGG) was used as the repair material for pure cement mortar, the interphase had a porous structure, which contained a rich amount of Ca(OH)_2_ and ettringite crystals and was detached from the structure after the pure concrete mortar dried out and shrank. Scanning electron microscopy (SEM) results demonstrated that repair using fly-ash mortar did not exhibit characteristics of calcium hydroxide or ettringite in the interphase, suggesting that fly ashes alleviate unfavorable microstructural characteristics in the interphase. The concrete–FGG interphase comprised compact C–S–H, and its strength increased slowly in the early stage but significantly in the long term. When fly ash was added, the structure exhibited its highest bond strength, and the decreased number of cracks and amount of carbonization caused dry shrinkage [5,6,7]. Geopolymer concrete reduces the environmental damage that carbon dioxide, which is abundantly produced in the manufacturing of Portland cement, causes to the environment. As a result, it is the most environmentally friendly material available to replace concrete. Ground-granulated blast-furnace slag (GGBFS) is an amorphous glass polymer. It is a hydraulic, cement material, and its chemical components are similar to those of concrete. Kuo et al. [8] used GGBFS to produce cementless concrete and discovered that its early strength was mainly provided by two sources: the main hydration products (i.e., ettringite, calcium hydroxide, and tobermorite gel) and the unhydrated clinker minerals. Adding fly ashes and concrete to geopolymers resulted in a similar reaction; it increased the strength and durability of FGG in the later stage, thus decreasing dry shrinkage [9]. Studies have suggested that fly ash-based geopolymers mixed with alkali excitation materials in an alkali metal solution could harden at room temperature [10] and demonstrated properties of high strength, bond strength, and durability to chloride [11]. The substantial dry shrinkage, cracks, and carbonization of FGG [12] can be overcome to achieve a longer lifespan for structures. Kuo et al. [13] identified that desulphurization slag powder (DS-P) and GGBFS can be used as new environmentally friendly noncement-based binding materials. Fernando et al. [14] used tungsten mine-waste geopolymers to repair concrete and revealed that geopolymers were the most economical of the studied repair materials for concrete structures. Alanazi et al. [15] used a metakaolin-based geopolymer to repair a new type of road surface and revealed that the metakaolin-based geopolymer exhibited favorable early strength, with 80% of the strength across the first 28 days gained in the first three days, and the favorable performance of this geopolymer was demonstrated in pull, split tensile, erosion, and comprehensive tests. Tanakorn et al. [16] used a high-calcium fly ash geopolymer mortar containing Portland cement as the repair material for concrete and discovered that it exhibited higher bond strength and bending stress than that of commercial repair materials. Ding et al. [17] using blast furnace slag and fly ash as raw materials, inorganic polymer was used as a repairing material for concrete structures, and the basic high viscosity and good working ability of blast furnace slag and fly ash were utilized. Using a molar ratio of SiO_2_ /Na_2_ base activator to find the optimum substitution ratio, the concrete substrate bonded with the geopolymer slurry. Compressive strength tests have shown that repair rates of up to 120% can be achieved. By comparison with Portland cement, it can be proved that the slag/fly ash base polymer slurry has a good potential for further engineering development in the future. To analyze the damage differences between FGG-repaired concrete and common concrete, the acoustic emission (AE) technique can be used as a monitoring tool in destructive tests. The AE technique is widely applied in nondestructive tests of metal materials, nonmetal materials, and engineering structures, in addition to being used to monitor the crack propagation properties in the fracture process of concrete [18,19]. In the AE technique, piezoelectric sensors are used for stress-wave monitoring and the received electromagnetic waves are converted into electrical signals. The fracture properties and brittleness of concrete are influenced by the inner brittleness of the material itself and the specimen condition settings [20]. The crack noise can be detected using the AE technique, which receives signals during the cleavage process in material bonds [21]. Accordingly, the AE technique is a tool that facilitates the monitoring and quantification of the damage to a specimen. 

The results of the aforementioned studies have demonstrated the potential of geopolymers to be used as repair materials. This study examined the effectiveness of FGG as a repair material, and various fly-ash substitution rates were used in FGG for these tests. The different combinations were then tested using slant shear and split tensile tests to establish a reference for using FGG as a repair material in the future.

## 2. Test Design

The production process of the specimens comprised two stages: the concrete was first produced and left to stand for 28 days and then repaired using FGG. The specimens were produced in molds and cured in water the day after the molds were removed. For the first part of the concrete specimen to be repaired, the model used in the pouring was the model that the institute built according to the specifications, and the changes were made at different angles. In the second stage, the specimen to be repaired was placed. The traditional model was carried out for repair. The compositions and properties of the concrete are displayed in Table 1, and the compositions of FGG are displayed in Table 2. In this study, the compositions of FGG were changed to achieve the required properties of repair materials under numerous conditions by adjusting the fly-ash substitution rate, liquid–solid ratio(L/S), and repair angle. Subsequently, the effectiveness and mechanisms of the various approaches in which FGG was used as the repair material were examined.

### 2.1. Material Properties

#### 2.1.1. GGBFS 

The GGBFS materials used in this study were provided by CHC Resources Corporation (Kaohsiung, Taiwan). The material had a fineness of 4207 cm^2^/g and a specific gravity of 2.29 g/cm^3^, meeting CNS 12549 of the Chinese National Standards.

#### 2.1.2. Fly Ash

The class F fly ashes used in this study were produced by a Taiwan Power Company thermal power plant (Kaohsiung, Taiwan). The fly ashes exhibited a fineness of 3818 cm^2^/g and a specific gravity of 2.16 g/cm^3^.

#### 2.1.3. Cement

This study used the type Ⅰ cement produced by the Southeast Cement Corporation (Kaohsiung, Taiwan), and it met the ASTM C150 Portland cement type I quality standard.

#### 2.1.4. NaOH

The solid NaOH used in this study was produced by the Zhong Ke company (New Taipei City, Taiwan), and exhibited a specific gravity of 0.598 g/cm^3^. Before the experiment, the sheet-shaped NaOH was mixed with deionized water to prepare a NaOH solution with a concentration of 14 M. 

#### 2.1.5. Sodium Silicate 

This study used the number three sodium silicate solution (37 °Bé) provided by Rong Xiang Industrial Co., Ltd. (Taoyuan, Taiwan).

#### 2.1.6. Course Aggregate

The study used natural rubble collected from Ligang, Pingtung in Taiwan. The 3/4” (9.6 mm) rubble exhibited a saturated-surface-dry (SSD) specific gravity of 2.71 g/cm^3^ and a water absorption capacity of 1.93%; moreover, the 3/8” rubble exhibited an SSD specific gravity of 2.65 g/cm^3^, water absorption capacity of 1.76%, and maximum grain size of 19 mm.

#### 2.1.7. Fine Aggregate

The study used natural sand collected from Ligang, Pingtung in Taiwan. The sand exhibited a fineness modulus of 3.06, an SSD specific gravity of 2.65 g/cm^3^, and a water absorption capacity of 1.93%.

### 2.2. Specimen Production

The FGG specimen production process was as follows. First, the GGBFS was dry-mixed with fine aggregate for 30 s, and then the dry mixture was mixed with an alkali activator for 120 s. After the mixture was left to stand for 15 s, the inner surface of the mixing container was scraped to move the mixed material to the center of the mixing container, and the components were subsequently mixed for 90 s. Next, the FGG was placed in the mold and then rammed and knocked to reduce air voids. After this step, the specimen mold was covered with cling film to reduce water loss. The specimen was demolded after it was air-hardened for 24 h, and then the specimen was cured in a water tank until the desired age was reached.

## 3. Test Methods

### 3.1. Slant Shear Test

**Figure 1:**
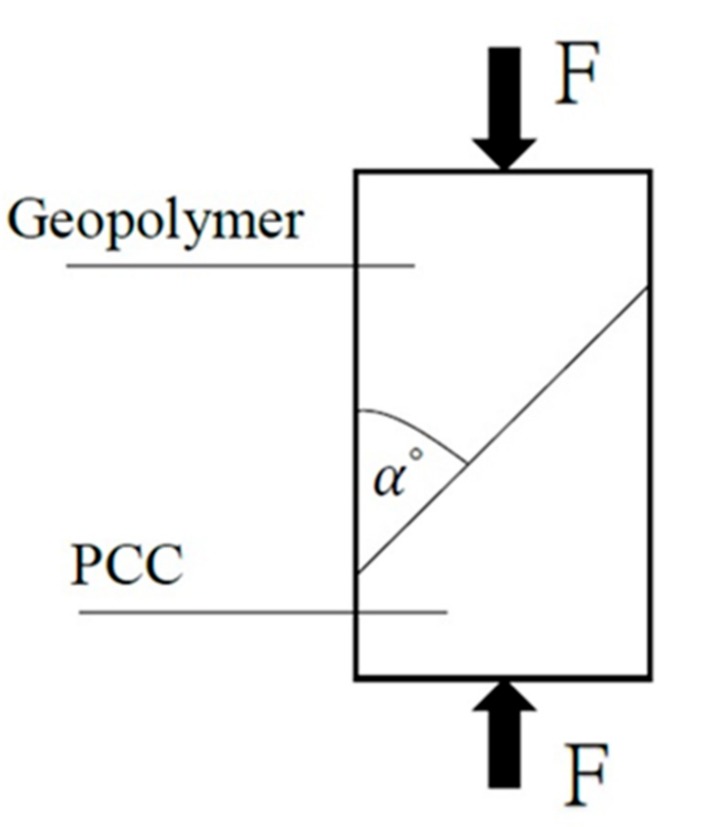
FGG slant shear test repair schematic.

A cylindrical specimen with a respective diameter and height of φ10 cm × 20 cm was produced based on ASTM C882 [22], cured in water until it reached the age of 28 days, and tested after its compressive surface was flat-lapped. The repair angle (α) of the cylindrical specimen was set at 45° and 60° for the slant shear test. This enabled the testing of the shear failure strength of the FGG-repaired concrete to assess the effectiveness of the repair. The calculation of the slant shear test was based on Equation (1), as demonstrated in Figure 1.

(1) $$\sigma \;＝\;\frac{F}{A}$$

where σ = slant shear test (MPa), F = maximum load of specimen (N), A = test area (mm^2^).

### 3.2. Split Tensile Test

**Figure 2:**
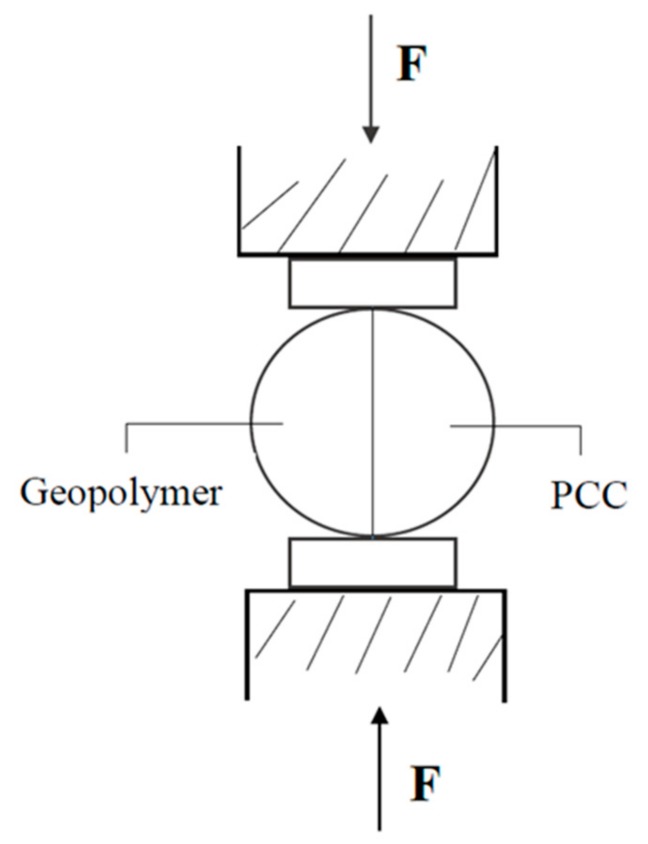
Split tensile test after repair.

A cylindrical specimen with a diameter and height of φ10 cm × 20 cm was produced based on Alanazi [15] and ASTM C496 [23]. The split tensile test employed was a standardized method for testing the tensile strength of cylindrical specimens, and was used to assess the bond strength between concrete and FGG. The split tensile test calculation was based on Equation (2), as demonstrated in Figure 2.

(2) $$T\;=\;\frac{2F}{\pi \mathrm{Ld}}$$

where: T = split tensile test (MPa), F = maximum load of specimen (N), L = length of test body (mm), d = specimen diameter (mm).

### 3.3. Applying the AE Technique to the Split Tensile Test

The AE technique was incorporated in the split tensile test to monitor the loading process of the FGG-repaired concrete specimen. The establishment of the relationship among the data of event, energy, and indirect tension strength facilitated real-time monitoring of the energy release during the fracture process. As a result, the fracture severity could be quantified.

### 3.4. Microstructural Characteristics

The study used ESEM (FEI Quanta 200 Environmental Scanning Electron Microscope) to observe the FGG–concrete interphase between the original and the new material, which could be used to predict the different factors that influenced the test. 

## 4. Results and Discussion

### 4.1. Slant Shear Test

**Figure 3:**
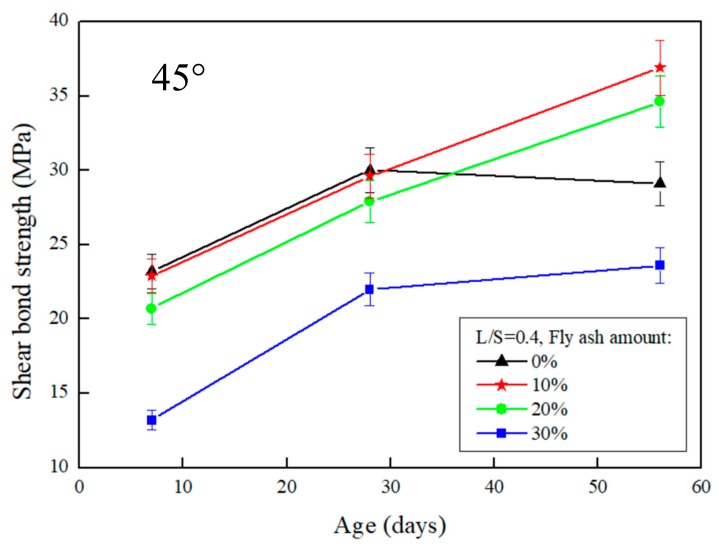
The effect of different fly-ash amounts on the slant shear test of FGG when L/S = 0.4 and repair angle was 45°.

**Figure 4:**
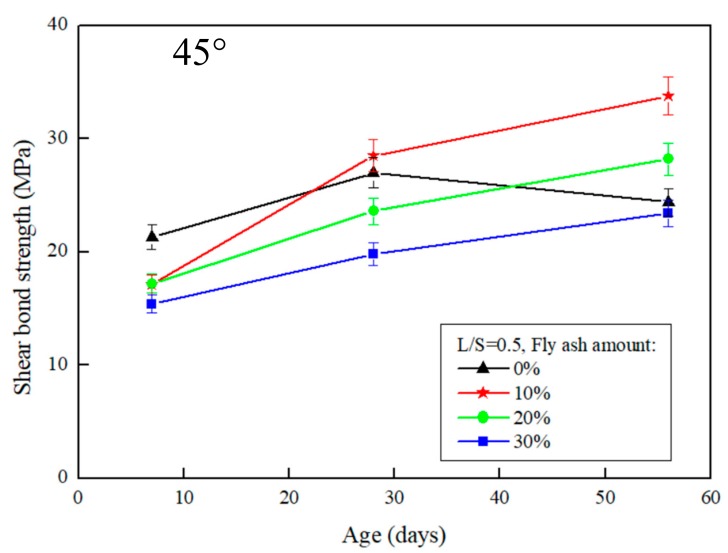
The effect of different fly-ash amount on the slant shear test of FGG when L/S = 0.5 and repair angle was 45°.

**Figure 5:**
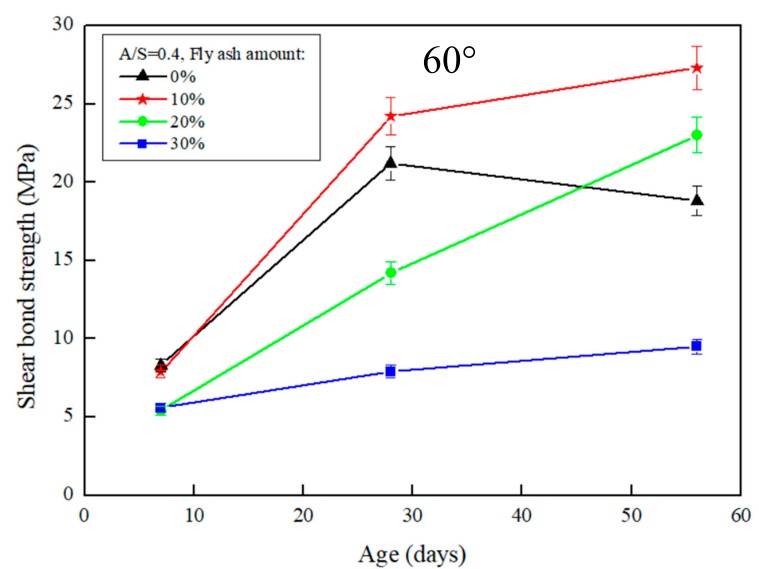
The effect of different fly-ash amount on the slant shear test of FGG when L/S = 0.4 and repair angle was 60°.

**Figure 6:**
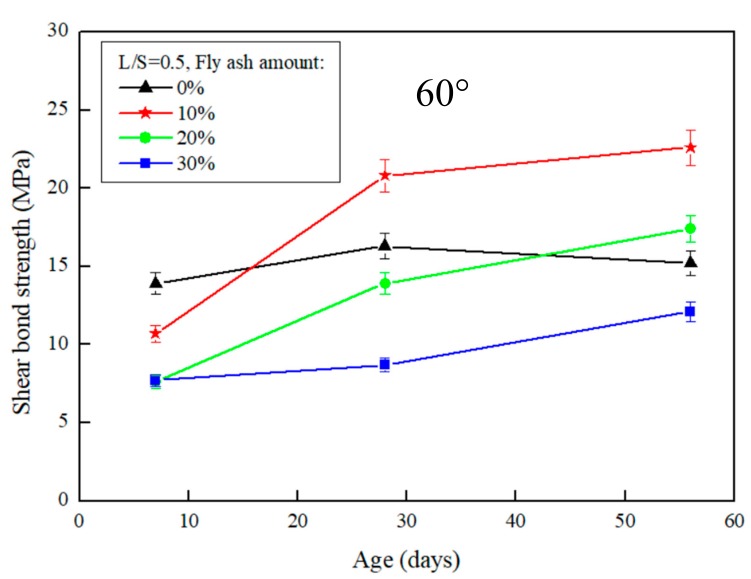
The effect of different fly-ash amount on the slant shear test of FGG when L/S = 0.5 and repair angle was 60°.

The results of the slant shear test after the repair using FGG are presented in Figure 3 and Figure 4. When the repair angle of the specimen was 45°, the liquid–solid ratio was between 0.4 and 0.5, and the shear strength was 36.9–33.8 MPa. When the repair angle of the specimen was 60° (Figure 5 and Figure 6), the liquid–solid ratio was between 0.4 and 0.5, and the shear strength was 27.3–22.6 MPa. These results indicate that the shear strength increased with an increase in the liquid–solid ratio but was decreased after repair. The main reason is that when the liquid–solid ratio increases, the use of water increases, thus reducing the concentration of OH^−^ ions in the alkali solution, impeding the formation of C–S–H gels, and decreasing the strength of the repaired material. A similar trend can be observed in Figure 3, Figure 4, Figure 5 and Figure 6. The fly-ash substitution rate of 10% exhibited a favorable repair effectiveness because an appropriate amount of fly-ashes increased the compactness of a specimen and slowed down the dry shrinkage caused by hot fissures. The repair strength of the studied fly-ash substitution rates was in the order of (most advantageous to least) 10%, 20%, 0%, and 30%. With a 0% fly-ash substitution rate, the repair strength decreased after 56 days, corresponding to the results presented in the study by Lee et al. [24], in which the average pore size of FGG was smaller than that of the ordinary Portland cement specimen. This indicates that an increase in small pores leads to high shrinkage, resulting in subtle cracks in FGG as well as a decrease in the flexibility modulus and long-term compressive strength, thus demonstrating an ineffective repair. Fly-ashes exhibited low activity with a 30% substitution rate, leading to this fly-ash value achieving the lowest strength of the tested substitution rates. 

**Figure 7:**
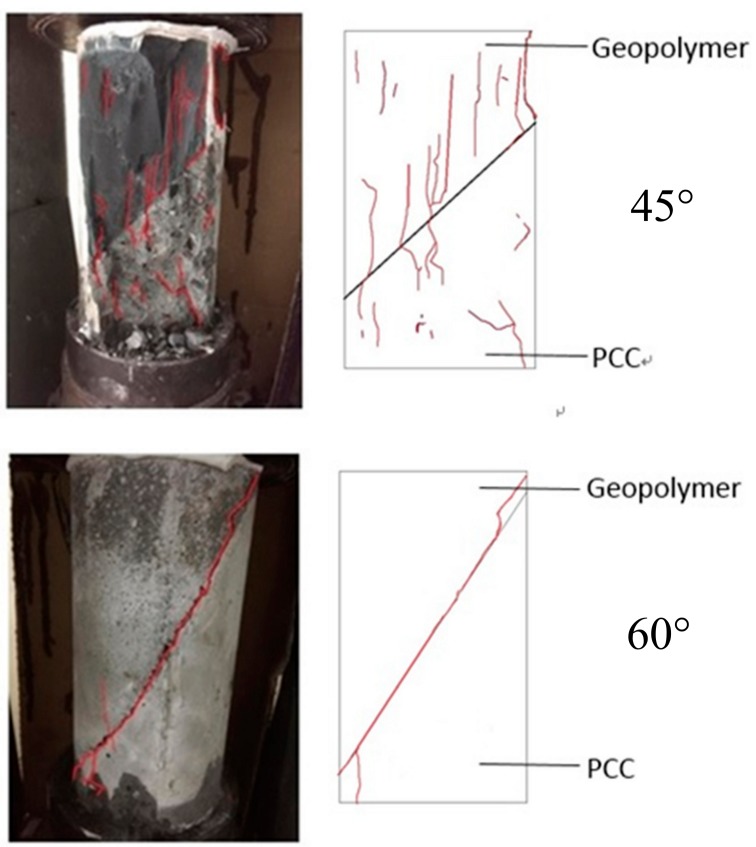
FGG repair angle when the damage pattern is different. (**a**) 45-degree angle cracked; (**b**) 60-degree angle sliding damage.

Regarding the angles (Figure 3 and Figure 5), the shear strength was 36.9–27.3 MPa when the slant shear angle was 45° and 60°. According to the results, the confined compression strength at the repair angle of 45° was superior to that at the repair angle of 60°. Lyu [25] conducted a slant shear test of concrete repairs and discovered that the shear strength was slightly reduced when the repair angle was 55° and 60°, thus confirming 55° as the critical mezzanine of sliding damage. This is consistent with the results of this present study, which posited that the difference in repair performance was caused by damage modes. When the repair angle was 45°, the damage comprised mostly cracks; specifically, the cracking interface ran through the repair material to the bottom concrete base, as demonstrated in Figure 7a. When the repair angle was 60°, the damage was mostly sliding damage; specifically, the breaking point was in the interphase, as demonstrated in Figure 7b.

### 4.2. Split Tensile Test

**Figure 8:**
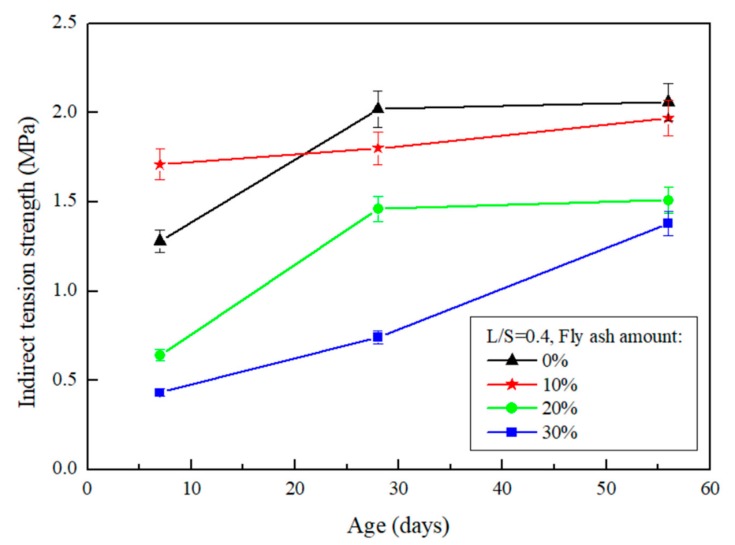
The effect of different fly-ash amount on FGG split tensile test when L/S = 0.4.

**Figure 9:**
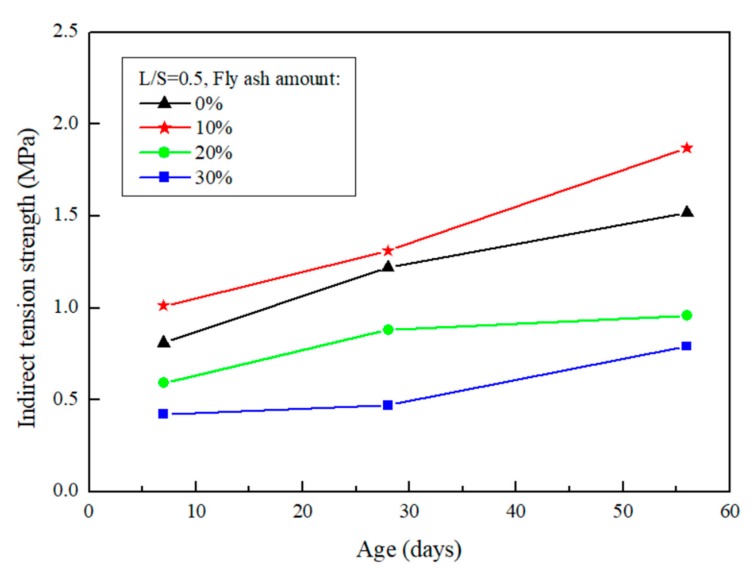
The effect of different fly-ash amount on FGG split tensile test when L/S = 0.5.

The results of the split tensile test after the repair using FGG are presented in Figure 8 and Figure 9. When the liquid–solid ratio was between 0.4 and 0.5, the split tensile test was 1.97–1.87 MPa, indicating that the increase in the liquid–solid ratio resulted in a decrease in tensile strength. Alanazi et al. [15] suggested the same destruction mechanism, and noted that all adhesive failures caused by the destruction mode occurred in the interphase. This team used the split tensile test to calculate the bond strength of a repair, and the results revealed that an increase in the liquid–solid ratio led to a decrease in FGG strength. The split tensile test was used in the present study to analyze the repair of a weak surface to obtain tensile strength data and then determine the compatibility. The results revealed that when the liquid–solid ratio increased, the tensile strength decreased. This may be because the liquid–solid ratio resulted in a decreased FGG strength, thus causing an imbalance between the strength of the two materials and leading to failure of the repair materials and decreased repair effectiveness.

When the fly-ash substitution rate was 0%, the repair strength was favorable in the early stage, whereas the rate of strength growth was decreased in the later stage. When the fly-ash substitution rate was 10%, although the rate of strength growth in the first 7 days was slow, it exhibited a higher strength in the later stage. This was because fly-ashes increase the compactness of a specimen and alleviate the dry shrinkage caused by hot fissures. When the fly-ash substitution rate was above 20%, the strength growth of the repaired structure was slow and substantially decreased. This is because fly-ashes exhibit low activity and slow developmental activity.

### 4.3. Application of AE in the Split Tensile Test to Monitor the Failure of the Specimen

**Figure 10:**
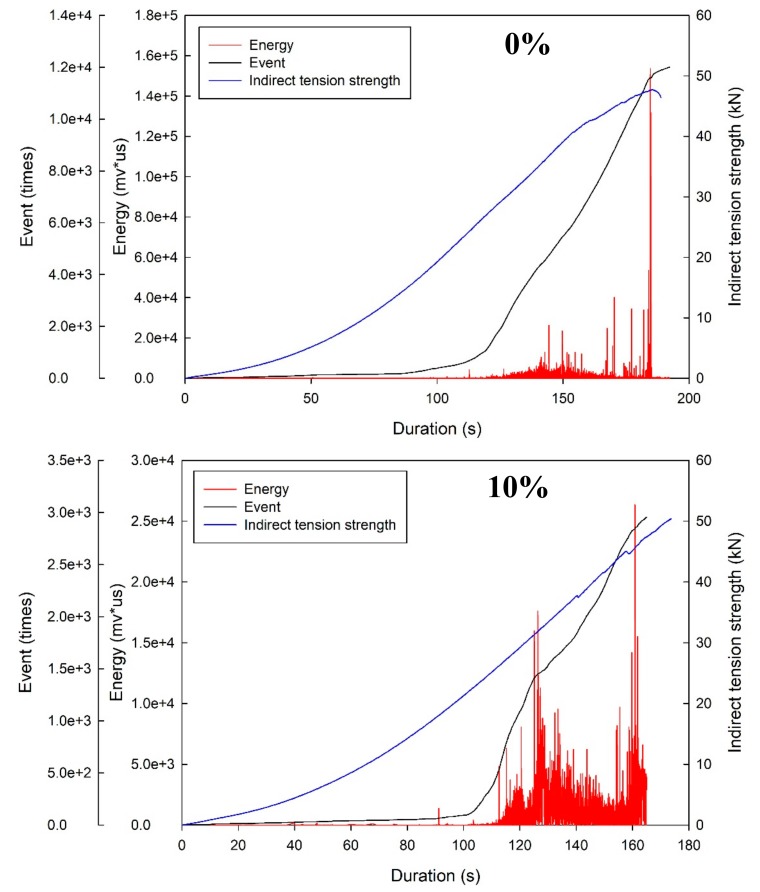

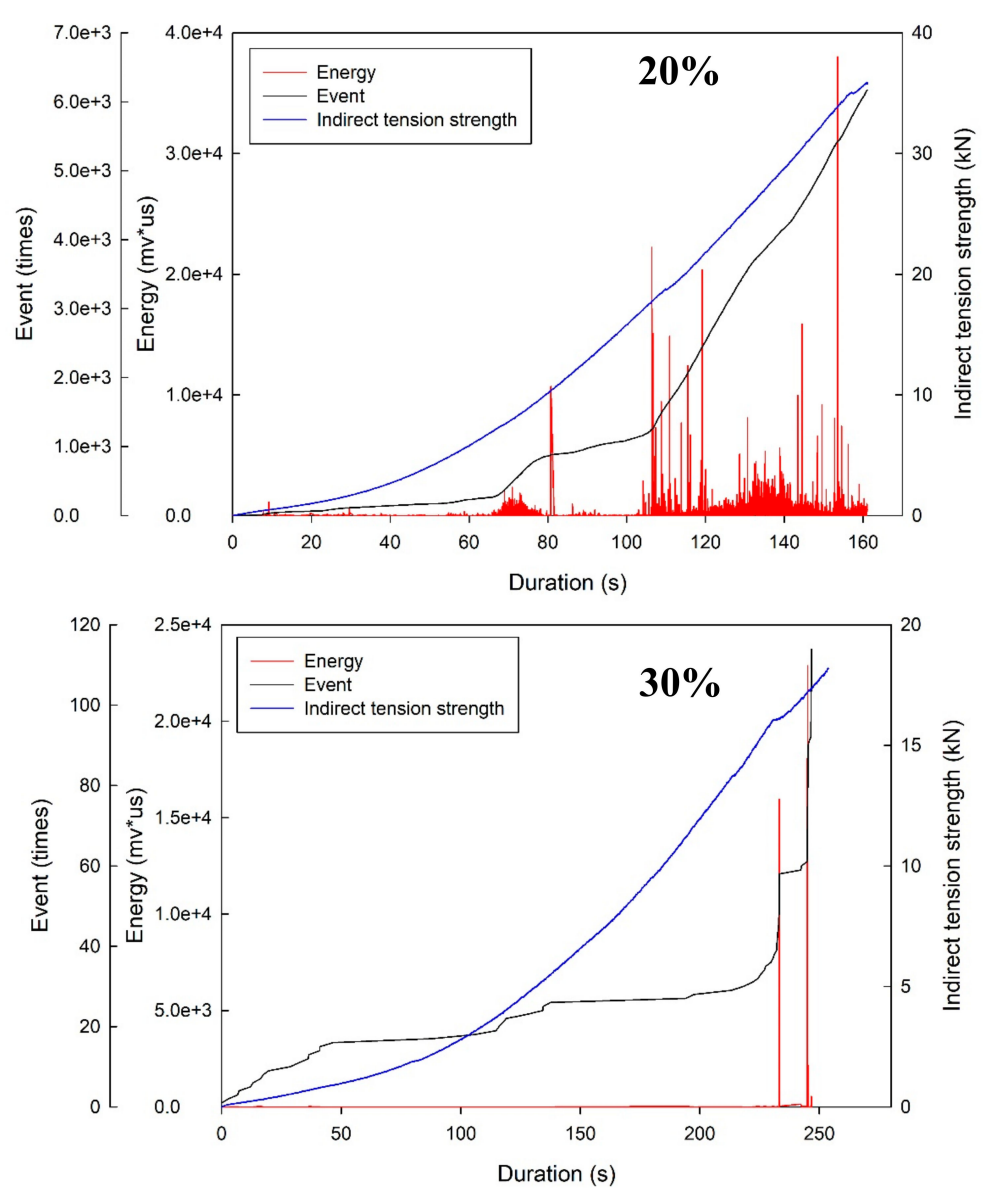
Application of acoustic emission (AE) in the split tensile test to monitor the failure of the specimen. (**a**) Fly-ash replacement 0%; (**b**) Fly-ash replacement 10%; (**c**) Fly-ash replacement 20%; (**d**) Fly-ash replacement 30%.

**Figure 11:**
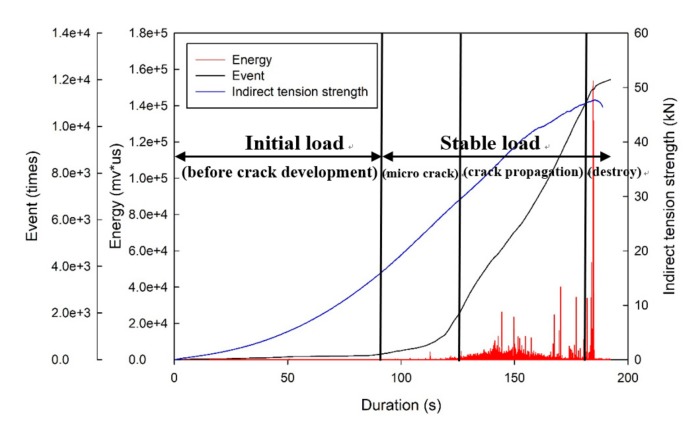
Application of AE in the split tensile test to monitor the destruction stage.

This study incorporated AE in the split tensile strength test to analyze the process from loading to damage, and the results were divided into three stages for analyses. The frontend stage ranged from the beginning to the first noticeable energy peak, the middle stage ranged from the first energy peak to 80% of the maximum load, and the backend stage was after the end of the middle stage. The test was used to record the energy release during the damage and was one of the methods used to test damage modes. The results are presented in Figure 10. The frontend stage contains considerable sounds of friction among the specimen caused by cracks. At approximately 80% of the highest loading, a noticeable large energy occurred, and these damages were all classified as no-warning damage. Thus, the four-stage damage behavior could be identified as follows: before the development of crack, microcrack generation, crack propagation distribution, and disruptive separation, as displayed in Figure 11. The stage before the development of cracks is when the load is less than the maximum load, which is classified as the stable condition; the microcrack generation stage is when the load reaches the maximum level and microcracks start to grow but the main crack does not; and the crack propagation distribution stage is when the external load reaches a critical level and the main crack starts to grow.

Using the same analysis methods applied by Geng et al. [26], the present study revealed that the damage characteristics of a concrete specimen under loading conditions was based on event and energy because they revealed the frequency of activity and energy release using AE. Based on a comparison between the present study and that conducted by Chen [27], the similarities between these studies are as follows: (1) in the initial load stage, almost no energy release occurred; (2) when the loading condition reached 80% of the maximum load, the AE energy became more active; and (3) when the coexistence of the highest energy and disruptive separation was monitored using the AE technique, the damage was classified as damage without warning.

### 4.4. Damage Mode

**Figure 12:**
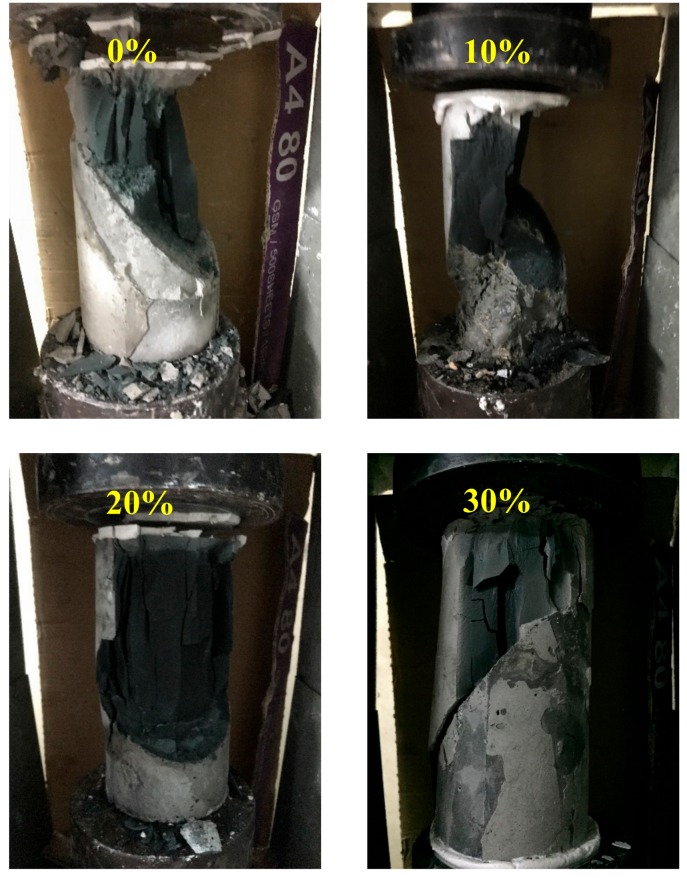
The slant shear test failure mode for different fly-ash amounts when L/S is 0.4. (**a**) Fly-ash replacement 0%; (**b**) Fly-ash replacement 10%; (**c**) Fly-ash replacement 20%; (**d**) Fly-ash replacement 30%.

This study employed a repair angle of 45° and a slant shear test to determine the damage behavior of FGG. The results of the study conducted by Lin [28] revealed that the compressive strength under a liquid–solid ratio of 0.4 was higher that under a liquid–solid ratio of 0.5. The sheet-shaped FGG after damage is illustrated in Figure 12. The 0–20% fly-ash substitution rates resulted in cracks in both the FGG and concrete; a cracking interface running through the repair material to the bottom concrete base was observed. The 30% fly-ash substitution rate produced sliding damage; that is, the breaking point was solely in the interphase.

### 4.5. Microstructural Characteristics

**Figure 13:**
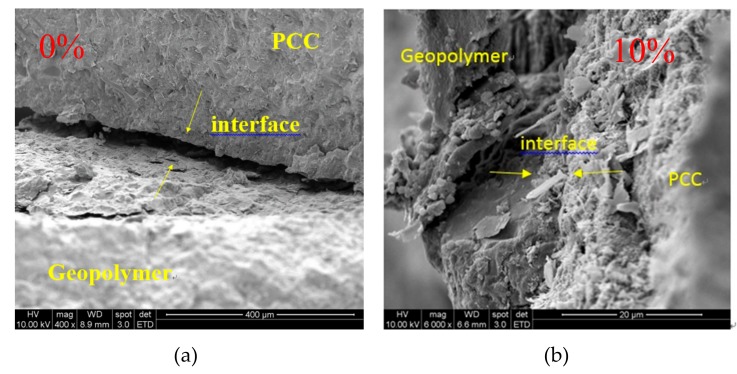

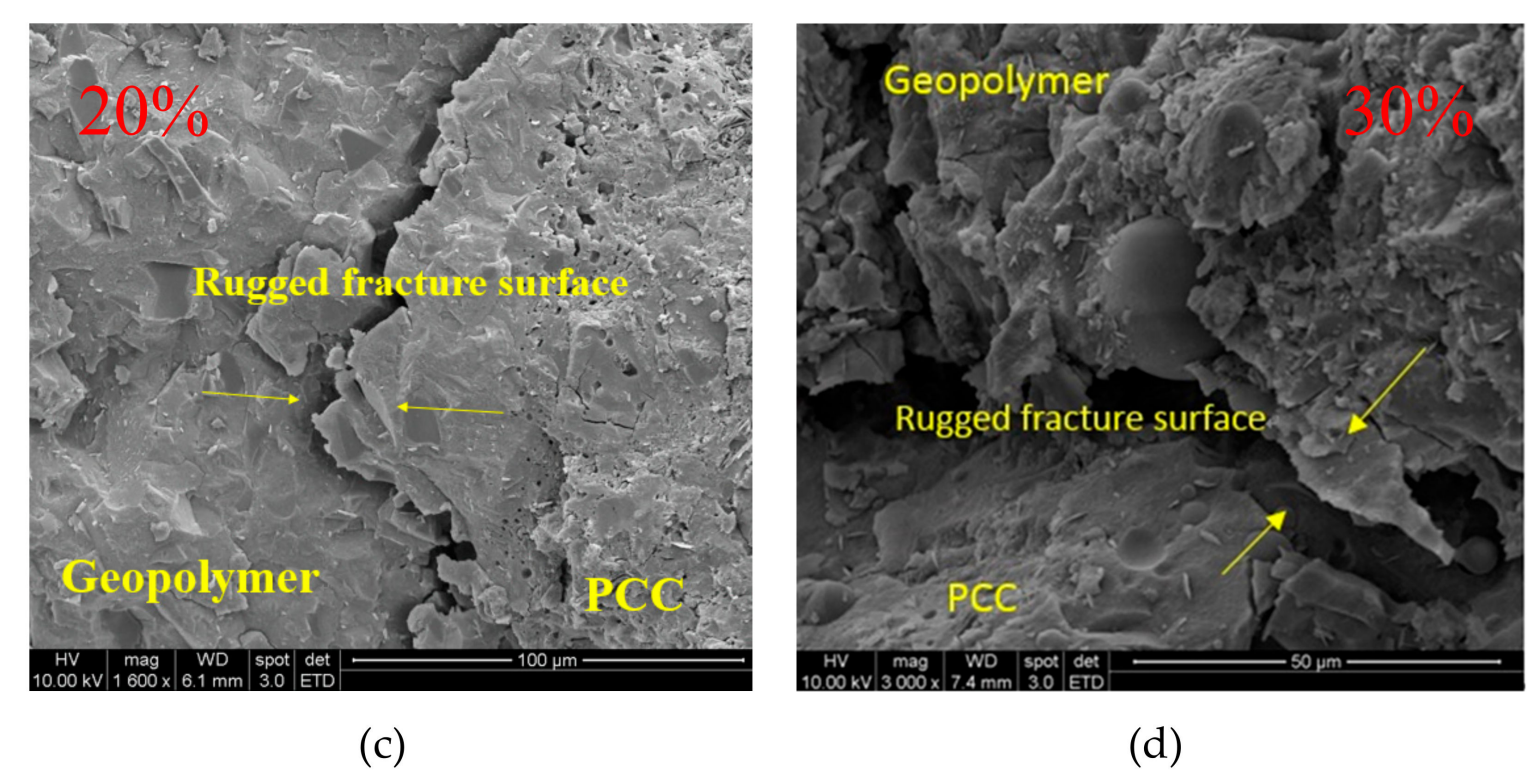
The microstructural characteristics of interphase when FGG was repaired with PCC. (**a**) Fly-ash replacement 0%; (**b**) Fly-ash replacement 10%; (**c**) Fly-ash replacement 20%; (**d**) Fly-ash replacement 30%.

**Figure 14:**
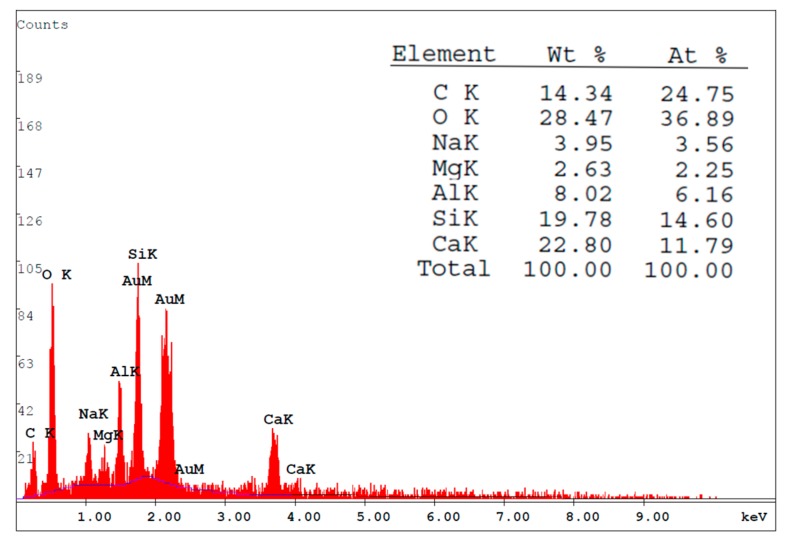
The energy-dispersive X-ray spectroscopy (EDX) component analysis of interface interphase when FGG was repaired with PCC.

This study used SEM to observe the interface between the concrete and the FGG, that is, the FGG–concrete interphase, as shown in Figure 13. The shape of the microstructural FGG was irregular. When the fly-ash substitution rate was 0% (Figure 13a), through SEM, the FGG–concrete interphase was revealed to be relatively clear, which might have been because the dry shrinkage of GGBFS resulted in poor bond strength. When the fly-ash substitution rate was 10% (Figure 13b), the FGG–concrete transition zone contained more C–S–H like hydration products than the other fly-ash conditions. When the fly-ash substitution rate was 20% (Figure 13c), there will be hydration products in the interphase, and there was no obvious hydration. When the fly-ash substitution rate was 30%, as displayed in Figure 13d, the cracks were larger in the FGG–concrete interphase, which may have been caused by the incomplete reaction of fly-ashes. Additionally, the imbalance of stress led to poor bond strength. The abundance of hydration products in the FGG–concrete interphase corresponds to the results obtained by Li et al. [4], Arnaud and Stephen [29], Sadowskki et al. [30], Khedmati et al. [31], Ivana and Tomas [32], Marjanovic et al. [33]. The slant shear strength and split tensile strength analyses in this present study revealed that the fly-ash substitution rate of 10% led to a higher bond strength than the other fly-ash conditions. Therefore, the study focused on the 10% fly-ash substitution rate and employed energy-dispersive X-ray spectroscopy (EDX) to analyze the components of the FGG–concrete interphase and the influence of bong strength. The use of EDX for analysis was in accordance with the research conducted by Li et al. [2]. The results of this analysis in the present study are presented in Figure 14. These results revealed that because geopolymers contain a substantial amount of Si^4+^ and Al^3+^, the bond mechanism reacted with the Ca(OH)_2_ on the surface of the concrete. The increase in Ca^2+^ counterbalanced the negative charge of Al^3+^, thus resulting in an increase in hydration products in the FGG–concrete interphase and an increase in bond strength.

## 5. Conclusion

1. The slant shear strength and split tensile strength results indicated that the bond strength of the FGG repair material decreased with an increase in the liquid–solid ratio, and a favorable repair effectiveness could be achieved when the fly-ash substitution rate was 10%.
2. The damage modes revealed that greater cracking noise was produced when the geopolymers were damaged, and these polymers became sheet-shaped after damage was inflicted; therefore, FGG has low brittleness and fatigue resistance.
3. This study incorporated the AE technique into monitoring the damage behavior of the repaired structure, and demonstrated that a higher bond strength produced a higher energy activity in the middle stage, confirming that AE energy is correlated with bond strength.
4. Using SEM to observe the microstructural characteristics of this structure, this study revealed that the hydration products in the interfacial interphase were more noticeable when the fly-ash substitution rate was 10% compared with when it was 30%, revealing that the influence of bond strength was highly related to the hydration products present in the interfacial interphase.
5. FGG through the slant shear test, split tensile test found that the L/S increased bond strength will drop, and fly-ash amount of 10% can get better repair effect.
6. From the failure mode, it is known that there is a large burst of sound when the geopolymer is destroyed, and the failure form is tile-like, so the FGG has the characteristics of low brittleness and poor fatigue resistance.
7. We introduced AE to monitor the damage behavior. When the bond strength was better, the energy of the middle part of the sound signal was obviously more active, and it was found that the acoustic energy had a correlation with the bond strength.
8. It is found that the influence of bond strength is closely related to the product of the interface interphase when the hydration product of the interfacial interphase is more obvious when the fly-ash amount is 10%.

## Figures and Tables

**Table 1:** Concrete proportion and properties to be repaired.

Components	PC (kg/m^3^)	Aggregates (kg/m^3^)		Water (kg/m^3^)	Properties of PCC	
		Fine	Coarse		fc’ (MPa)	ft’ (MPa)
Proportions	277	886	974	201	28.9	7.8

fc’ = 28 days compressive strength (ψ = 10 cm, H = 20 cm); ft’ = 28 days bending strength (10 cm × 10 cm × 35 cm); PC = Portland cement; PCC = Portland cement concrete

**Table 2:** Fly ash/ground-granulated blast-furnace slag geopolymer (FGG) variable.

Alkali Modulus	Alkali Equivalent	NaOH (M)	Bé	FA Amount (%)	L/S	Repair Angle (α)
1.0	8%	14	37°	0/10 or 20 or 30	0.4 or 0.5	45° or 60°
